# Numerical Assessment of a Metal-Insulator-Metal Waveguide-Based Plasmonic Sensor System for the Recognition of Tuberculosis in Blood Plasma

**DOI:** 10.3390/mi14040729

**Published:** 2023-03-25

**Authors:** Muhammad A. Butt

**Affiliations:** Warsaw University of Technology, Institute of Microelectronics and Optoelectronics, Koszykowa 75, 00-662 Warszawa, Poland; ali.butt@pw.edu.pl

**Keywords:** metal-insulator-metal waveguide, plasmonics, tuberculosis, blood plasma, surface plasmon polariton, mode converter

## Abstract

In this paper, a numerical analysis of a plasmonic sensor based on a metal-insulator-metal (MIM) waveguide is conducted for the detection of tuberculosis (TB)-infected blood plasma. It is not straightforward to directly couple the light to the nanoscale MIM waveguide, because of which two Si_3_N_4_ mode converters are integrated with the plasmonic sensor. This allows the efficient conversion of the dielectric mode into a plasmonic mode, which propagates in the MIM waveguide via an input mode converter. At the output port, the plasmonic mode is converted back to the dielectric mode via the output mode converter. The proposed device is employed to detect TB-infected blood plasma. The refractive index of TB-infected blood plasma is slightly lower than that of normal blood plasma. Therefore, it is important to have a sensing device with high sensitivity. The sensitivity and figure of merit of the proposed device are ~900 nm/RIU and 11.84, respectively.

## 1. Introduction

Plasmonics is an interdisciplinary field of science that deals with the study of plasmons—the collective oscillations of free electrons in a metal or semiconductor material [1]. Plasmonics has significant potential in various fields of research, including nanotechnology, biomedical engineering, and information technology [2,3]. Plasmonics provides a new way to control and manipulate light at the nanoscale, which has important implications for the development of new optoelectronic devices such as nanolasers, optical switches, and photovoltaic cells [4,5,6,7]. Plasmonics-based sensors can detect and measure minute changes in the refractive index of materials, making them valuable tools in areas such as medical diagnostics, environmental monitoring, and food safety [8].

A metal-insulator-metal (MIM) based plasmonic sensor is a type of plasmonic sensor that uses a sandwich-like structure composed of two metallic layers separated by a thin insulating layer [9]. The metallic layers typically consist of noble metals, such as gold or silver, while the insulating layer may be made of materials such as silicon dioxide, titanium dioxide, or aluminum oxide [10]. In a MIM-based plasmonic sensor, the metal-insulator interface acts as a resonant cavity that supports plasmon modes [11]. When light is incident on the sensor, the plasmons on the metal surface are excited and resonate at a specific frequency, resulting in a strong electromagnetic field that is highly localized near the metal-insulator interface. The presence of a sample on the sensor surface changes the refractive index of the insulating layer, causing a shift in the resonance frequency of the plasmons. This shift can be detected by measuring changes in the transmission, reflection, or absorption spectra of the sensor, providing information about the concentration and properties of the target analyte [12,13,14,15].

MIM waveguide-based plasmonic sensors have many advantages, including high sensitivity, high resolution, and the ability to detect small changes in the refractive index of the sample [16,17,18,19]. They can be used in various applications, such as chemical sensing, biosensing, and environmental monitoring [17,20,21,22,23,24]. However, they also have some limitations, including the requirement for specialized fabrication techniques and the difficulty of integrating them with other technologies, such as microfluidics or electronics [25].

Tuberculosis (TB) is primarily an infection of the lungs, but it can also affect other parts of the body such as the lymph nodes, bones, and kidneys. In rare cases, TB can affect the bloodstream, which is known as miliary tuberculosis. Blood plasma is the yellowish liquid component of blood that makes up about 55% of the total volume of blood. It is composed of water, proteins, electrolytes, hormones, and nutrients that are essential for various bodily functions such as transporting nutrients and oxygen to tissues, removing waste products, and regulating body temperature [26]. Blood plasma can provide valuable information for the early detection of a wide range of diseases. By analyzing the proteins and other molecules present in blood plasma, healthcare professionals can identify potential health issues and take appropriate measures to manage or treat them before they become more serious.

In the context of TB, blood plasma may contain TB bacteria in cases of miliary tuberculosis, where the bacteria have spread throughout the body via the bloodstream. However, the diagnosis of miliary TB is usually made through imaging tests such as chest X-rays, CT scans, or MRIs rather than through blood tests alone. Blood tests may be used to diagnose latent TB infection or active TB disease in the lungs. In this paper, we proposed a plasmonic sensor based on a MIM waveguide for the detection of TB in blood plasma. The refractive index of the TB-infected blood plasma is slightly lower than normal blood plasma which can be effectively accessed via a plasmonic sensor, and the sensitivity offered by the proposed device is ~900 nm/RIU.

## 2. MIM Waveguide-Based Plasmonic Sensor Design

**Figure 1:**
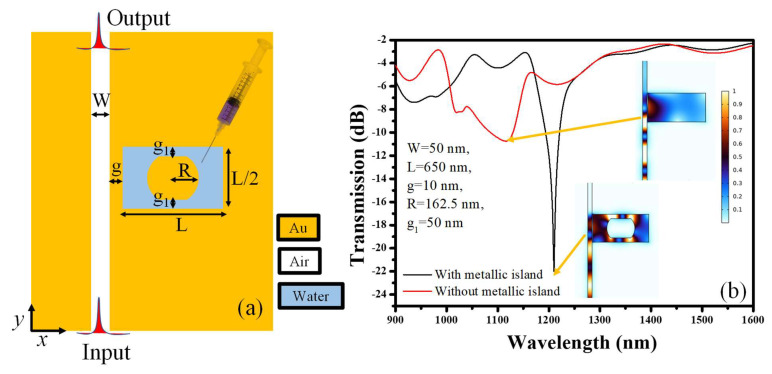
(**a**) Schematic representation of a MIM waveguide-based plasmonic sensor; (**b**) Transmission spectra of the sensor with a metallic island and without a metallic island.

The schematic representation of the sensor design is shown in Figure 1a. It comprises a MIM bus waveguide which is side coupled to a rectangular hollow cavity with a metallic island inside. This arrangement provides stronger field confinement as compared to a simple rectangular hollow cavity at resonance wavelength. A MIM waveguide is a type of waveguide used in the field of optics to guide and manipulate the propagation of light. It consists of a thin insulating layer sandwiched between two metal layers, forming a planar structure. The metal layers act as mirrors that reflect light back and forth within the waveguide, while the insulating layer acts as a dielectric that helps confine the light to the waveguide region. The waveguide is typically designed to have a subwavelength thickness, which allows for strong confinement of the light in the transverse direction. In our proposed sensor design, gold (Au) and air are selected as metal and dielectric materials to form a MIM waveguide. Au is biologically inert, meaning it does not react with body fluids or tissues. It is also non-toxic and non-allergenic, which further adds to its biocompatibility. Additionally, gold is resistant to corrosion, which is important for maintaining the structural integrity of medical devices. The Lorentz-Drude model is used to calculate the permittivity of Au [27], as indicated in Equation (1)

(1) $$\varepsilon =\varepsilon_{\infty }-\frac{\omega_{p}^{2}}{\omega^{2}+j\omega \gamma }$$

where $\varepsilon_{\infty }=9.0685$, $\omega_{p}$=135.44 × 10^14^ rad/s, and γ=1.15 × 1014 rad/s. The width of the MIM waveguide is denoted as W, which is fixed at 50 nm and supports the fundamental TM_0_ mode. Nanogaps can be designed using focused ion beam lithography (FIBL) [28] or E-beam lithography (EBL) [29], which can accomplish a better resolution of sub-10 nm. The side length of the rectangular cavity is denoted as L, and the width is expressed as L/2. The gap between the cavity and the MIM bus waveguide is labeled as g. As far as the metallic island is concerned, it has a radius (R) where a gap (g_1_) is introduced on two sides to allow the flow of light in the whole cavity. This metallic island provides strong light confinement in the narrow gaps, which enhances the sensitivity of the device. The transmission spectrum of the device with and without a metallic island in the cavity is shown in Figure 1b. The E-field is significantly enhanced in the cavity with a metallic island resulting in a sharp resonance dip with a high extinction ratio of −22 dB. The E-field enhancement is due to the narrow path between the island and the hollow cavity which confines the resonant wavelength of light around the island as can be seen in the inset of Figure 1b.

Many physics and engineering issues may be resolved numerically using the finite element method (FEM). Approximating the general behavior of the system entails breaking a complicated problem down into smaller, simpler segments, or “elements,” and then applying mathematical equations to each element. A set of algebraic equations that may be resolved numerically in FEM serve as an approximation for the continuous issue. The primary concept is to split the domain into a limited number of smaller subdomains, or elements, where the governing equations are approximated by a set of algebraic equations that can be solved by a computer. The piecewise polynomial function that satisfies the governing equations and boundary conditions within each element is the approximative solution produced by FEM. The transmittance and field mappings are modeled with the FEM using the COMSOL Multiphysics program. The sub-domains of the device design are broken down into triangular mesh components with a grid size of 5 nm, using the available computer capabilities. This makes it easier to provide reliable simulation results. It is ideal to build an open-bounded domain, or limit of the computing domain, where an electromagnetic wave travels without any reflection while evaluating electromagnetic wave issues. Scattering boundary conditions (SBC) are utilized to depict an open geometry at the borders of the FEM simulation window.

## 3. Device Optimization

It is always essential to optimize the geometric parameters of the device to obtain the finest transmission characteristics before utilizing it for sensing applications. The accuracy of a sensor depends on many factors, including the size, shape, and position of its cavity. By optimizing these geometric parameters, sensors can be designed to have higher accuracy and precision [30,31]. The sensitivity of a sensor is another important factor that can be improved through geometric parameter optimization. By adjusting the size and shape of a sensor’s cavity, its sensitivity to changes in the environment can be increased. The detailed geometric parameters used in the optimization are listed in Table 1.

**Figure 2:**
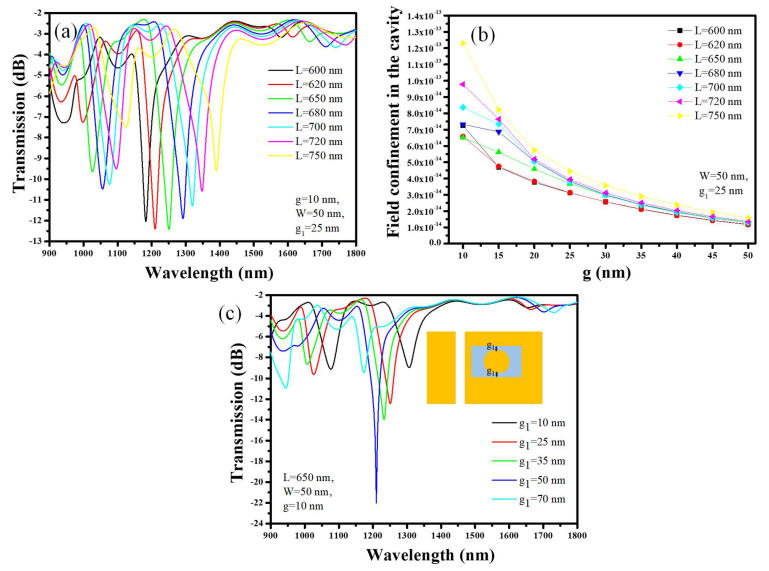
Device optimization: (**a**) Transmission vs. L, (**b**) Field confinement in the cavity versus g, (**c**) Transmission versus g_1_.

In the first step, the side length (L) of the rectangular cavity with the metallic island is varied between 600 nm and 750 nm. As a starting point, the remaining geometric parameters such as W, g, and g_1_ are fixed at 50 nm, 10 nm, and 25 nm, respectively. The resonance wavelength performs a redshift as L increases from 600 nm to 750 nm due to the increase in the effective length of the cavity, as shown in Figure 2a. This signifies that L is an important parameter to control the transmission spectrum in a specific range.

In the second step, the coupling gap (g) between the bus waveguide and the cavity is optimized. The remaining geometric parameters, such as W and g_1_, are fixed at 50 nm and 25 nm, respectively. Electric field confinement in a cavity refers to the ability of the cavity to trap and confine electromagnetic waves within its boundaries. This is achieved through a process called resonance, where the frequency of the electromagnetic wave matches the resonant frequency of the cavity. When an electromagnetic wave enters a cavity, it reflects back and forth between the walls of the cavity. As the wave reflects, it interferes with itself constructively, leading to a buildup of energy at the resonant frequency of the cavity. This results in a strong electric field within the cavity that is confined to the region between the walls. The strength of the electric field confinement in a cavity depends on several factors, including the gap between the bus waveguide and the cavity, the size and shape of the cavity, the material properties of the walls, and the frequency of the electromagnetic wave. From Figure 2b, it can be seen that the electric field confinement (Vxm) in the cavity at resonant wavelength decreases as g increases from 10 nm to 50 nm. Therefore, it is important to place the cavity as close as possible to the bus waveguide, depending on the resolution of the fabrication method. As mentioned earlier, the E-beam can provide a resolution of ~10 nm; therefore, we assume that 10 nm is the optimum value of g where maximum field confinement can be obtained. In the last step, gap (g_1_) between the upper and lower walls of the cavity and the metallic island is varied between 10 nm and 70 nm to enhance the field confinement in the cavity. As g_1_ increases, the resonance wavelength performs a blueshift due to a decrement in the effective index of the mode, and the maximum field confinement is obtained at g_1_ = 50 nm where the extinction ratio of −22 dB is obtained as shown in Figure 2c.

**Figure 3:**
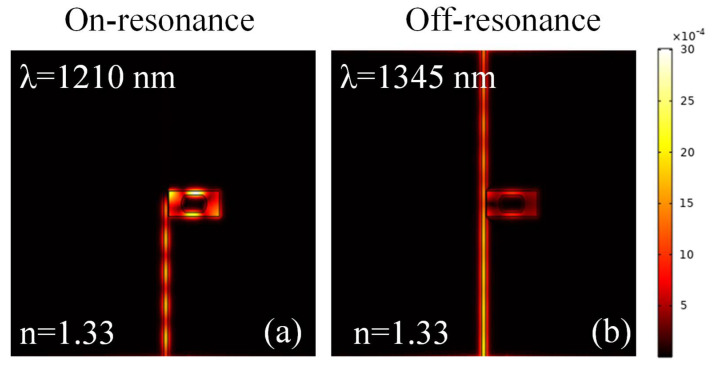
H-field distribution at (**a**) an on-resonance state and (**b**) an off-resonance state.

To visually analyze the behavior of light at different resonance states, it is important to plot the magnetic (H) field distribution in the sensor. The H-field distribution in the sensor in on-resonance and off-resonance states are shown in Figure 3a,b, respectively. This shows that the light is strictly confined in the cavity in the on-resonance state, leading to a sharp dip in the transmission spectrum, whereas in the off-resonance state, the light travels from the input port to the output port without coupling to the cavity. The geometric parameters used in the analysis are as follows: W = 50 nm, L = 650 nm, g = 10 nm, and g_1_ = 50 nm. Additionally, the cavity is filled with water, i.e., n = 1.33.

## 4. Refractive Index Range of Blood Plasma Infected with TB

Urine and blood tests are the first to show the effects of any illness on the human body. The refractive index profile of human blood and urine is a significant metric by which pathologists are intrigued. At three million deaths per year, or about five per minute, TB is still the biggest infectious illness responsible for high mortality among people. There are 8–10 million individuals affected by mycobacterium TB every year [32]. Data on the plasma’s refractive index from TB patients’ blood are presented by Reddy et al. [32]. Using Abbes’ refractometer, blood plasma’s refractive index is assessed. The experimental values are contrasted with those of blood plasma, which is typically found. Whereas the refractive index of plasma in normal blood is 1.351, that of tuberculosis patients’ blood ranges from 1.343 to 1.35, as shown in Table 2. As a result, when compared to normal blood, the refractive index values of tuberculosis blood are slightly lower.

**Figure 4:**
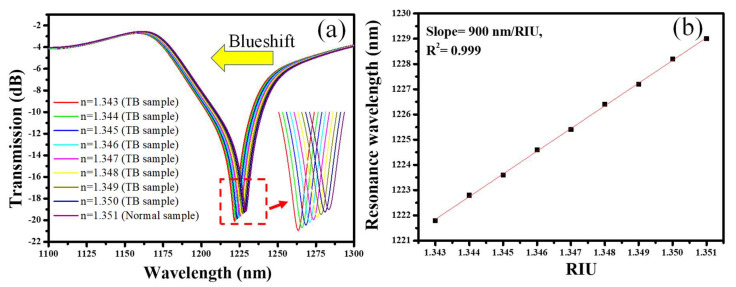
Sensing performance; (**a**) transmission spectrum of the sensor in the presence of normal and TB-infected blood plasma; (**b**) resonance wavelength versus RIU.

The transmission spectrum of the device is plotted for the normal and TB-infected blood plasma samples as shown in Figure 4a. As the refractive index of the TB-infected samples is lower than the normal plasma sample, the resonance dip performs a blueshift. The sensitivity of a sensing device is crucial because it determines the smallest detectable change in the quantity being measured that the sensor can detect. In other words, the sensitivity of a device indicates how responsive the sensor is to a small change in the physical quantity it is designed to measure. A sensor with higher sensitivity can detect smaller changes in the quantity being measured and can provide more accurate and precise measurements. For example, a highly sensitive biosensor can detect even small changes in refractive index, which is important in applications where even a small change in the refractive index of blood plasma can have significant consequences. The sensitivity of the refractive index sensor is calculated using Equation (2) [33]:

(2) $$\mathrm{Sensitivity}\;\left(S\right)=\frac{\Delta \lambda }{\Delta n}\;$$

where ∆λ and ∆n are the change in resonance wavelength and change in refractive index, respectively. Linear regression is a common technique used to fit a straight line to a set of data points to estimate the relationship between two variables. One of the key outputs of this technique is the slope of the line, which represents the rate of change in the dependent variable for each unit increase in the independent variable. By applying the linear fitting to the resonant wavelength versus the RIU graph, a slope of ~900 nm/RIU is obtained, as shown in Figure 4b.

The figure of merit (FOM) of a sensor is a quantitative measure of its performance or effectiveness in measuring a specific parameter. The FOM is typically used to compare different sensors or to evaluate the performance of a single sensor under different conditions. In general, a higher FOM indicates that a sensor is better at measuring the parameter of interest. However, it is important to consider the specific application and requirements of the measurement when selecting a sensor, as the optimal FOM may vary depending on the circumstances. The FOM of the sensor is calculated using Equation (3) [34]:

(3) $$\mathrm{FOM}=\frac{S}{\mathrm{FWHM}}$$

where S is the sensitivity and FWHM is the full width at half the maximum of the resonant wavelength. The FWHM of the resonance dip is ~76 nm, which means that the FOM of the device is ~11.84.

## 5. Integration of Dielectric Mode Converters to the Plasmonic Sensor

For several tasks, it is crucial to efficiently and compactly couple light from space to plasmonic devices. For instance, the relatively large footprint of photonic integrated circuits (PICs), which show tremendous promise in a variety of technologies such as mobile devices, wearable electronics, and the Internet of Things (IoT), poses a hurdle. The diffraction limit at optical wavelengths causes modern PIC devices to take up a lot of space (compared with state-of-the-art electronic circuits with their copper interconnects). Minimizing the footprint of the couplers and interconnects is crucial for the acceptance of PIC technology in many applications.

Direct coupling of light into a MIM waveguide can be achieved using a variety of techniques, such as optical fiber tapers, grating couplers, or near-field probes. These techniques can be used to couple light from a free-space beam to the MIM waveguide, or from a nearby waveguide or device. One of the challenges of direct coupling into a MIM waveguide is the mismatch between the optical mode in the free space or input waveguide and the SPP mode in the MIM waveguide. This mismatch can lead to losses or inefficient coupling of the light into the MIM waveguide. However, various design strategies and optimization techniques can be used to overcome this challenge, such as shaping the geometry of the waveguide, adjusting the thickness of the metal layer, or using adiabatic tapers [35].

Dielectric-to-plasmonic mode converters are used to convert a signal propagating in a dielectric waveguide into a plasmonic signal that can be transmitted through a plasmonic waveguide [36]. This type of converter is useful for integrating dielectric and plasmonic waveguides in nanophotonic circuits. Dielectric waveguides support modes that are primarily composed of electric and magnetic fields in the dielectric material. In contrast, plasmonic waveguides support modes that are primarily composed of SPPs, which are electromagnetic waves that propagate along the interface between a metal and a dielectric material.

**Figure 5:**
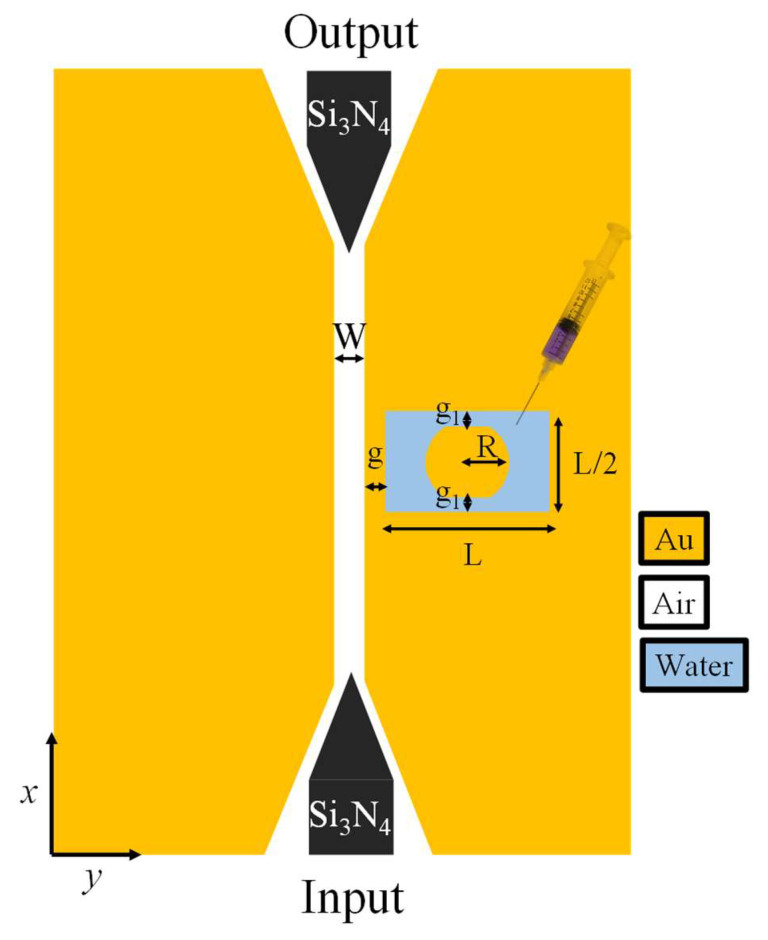
Schematic representation of the MIM waveguide-based plasmonic sensor integrated with dielectric mode converters.

For that reason, we have designed Si_3_N_4_ mode converters in the form of a tapered waveguide, as shown in Figure 5. One application of tapered waveguide mode converters is in integrated optics, where they are used to couple light between different waveguide modes in photonic integrated circuits. For instance, a tapered waveguide mode converter can be used to couple light between a single-mode waveguide and a multimode waveguide, or between two waveguides with different dimensions [36].

**Figure 6:**
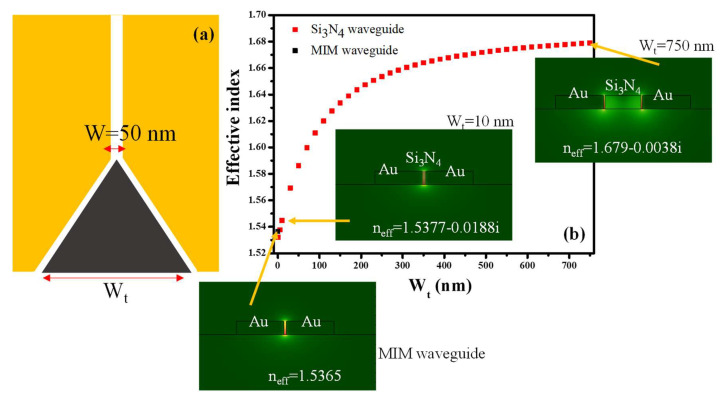
(**a**) Schematic representation of a mode converter integrated with a MIM waveguide; (**b**) Effective index versus tapered waveguide width. Inset shows the E-field distribution and effective index in the waveguide at a different position along the tapered waveguide.

Figure 6a shows the segment of the mode converter and the MIM waveguide, which we would like to analyze for effective index matching. The height and width (W_t_) of the Si_3_N_4_ waveguide are maintained at 280 nm and 750 nm, for single-mode operation, respectively. In the tapered segment, W_t_ reduces from 750 nm to 5 nm, and the tip of the tapered waveguide is located at the input of the MIM waveguide. It must be noted that the gap between the Au layer and the Si_3_N_4_ taper is maintained at 25 nm on both sides. The effective index of the whole tapered waveguide segment is plotted in Figure 6b. It can be seen that the effective index of the hybrid mode at W_t_ = 750 nm is 1.679–0.0038i, which reduces to 1.5377–0.0188i as W_t_ approaches 5 nm. Whereas the effective index of the MIM waveguide is ~1.5365, which provides an effective index match of 99.9%, resulting in the effective conversion of the dielectric mode to the plasmonic mode. Inset shows the H-field distribution and the effective index value of the mode in the tapered segment when W_t_ = 750 nm and W_t_ = 5 nm. Moreover, the H-field distribution in the MIM waveguide is also shown.

**Figure 7:**
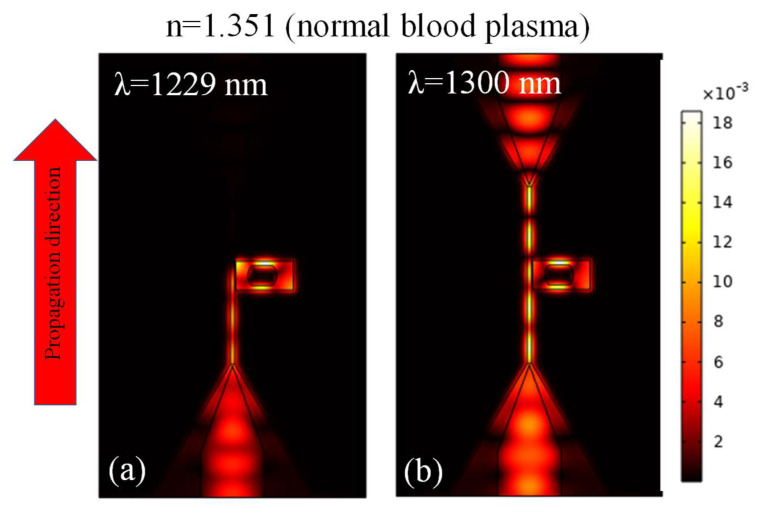
H-field distribution in the plasmonic sensor system at, (**a**) on-resonance state, (**b**) off-resonance state.

After integrating the mode converters in the MIM waveguide-based plasmonic sensor, the H-field distribution is plotted for the on-resonance state and off-resonance state, as shown in Figure 7a,b, respectively. The geometric parameters of the sensing system are as follows: W = 50 nm, L = 650 nm, g = 10 nm, g_1_ = 50 nm, and W_t_ = 750 nm.

## 6. Conclusions

In this paper, a plasmonic sensor based on a MIM waveguide is proposed and numerically analyzed via the finite element method for the detection of tuberculosis (TB) infected blood plasma. The size and shape of the micro-cavity in the plasmonic resonant sensors are quite crucial in obtaining a sharp resonance dip. Therefore, we suggested a hollow rectangular cavity with a metallic island. This configuration provides a resonance dip of −22 dB in the transmission spectrum. The refractive index of TB-infected blood plasma is slightly lower than that of normal blood plasma. As a result, a blueshift in the transmission spectrum is observed. The sensitivity offered by the proposed sensing device is ~900 nm/RIU. Additionally, Si_3_N_4_ mode converters are also embedded with the MIM waveguide plasmonic sensor for the efficient coupling of light. The mode conversion efficiency of the tapered waveguide is around 99.9%, which ensures smooth transformation from the dielectric mode to the plasmonic mode. We believe that this study will pave the way for the development of MIM waveguide-based plasmonic sensors for several eye-catching applications.

## Figures and Tables

**Table 1:** Geometric parameters of the device.

Geometric Variables	Value
W	50 nm (fixed)
g	10 nm to 50 nm
g_1_	10 nm, 25 nm, 35 nm, 50 nm, 75 nm
R	150 nm to 187.5 nm
L	600 nm to 750 nm

**Table 2:** The refractive index of normal and TB-infected blood plasma samples obtained from [32].

Sample	Refractive Index Value
TB-infected	1.343
TB-infected	1.344
TB-infected	1.345
TB-infected	1.346
TB-infected	1.347
TB-infected	1.348
TB-infected	1.349
TB-infected	1.350
Normal	1.351

## Data Availability

Not applicable.
